# Supplementary material for: Ectopic Expression of Plasmodium vivax vir Genes in P. falciparum Affects Cytoadhesion via Increased Expression of Specific var Genes
Source: Microorganisms. 2022 Jun 9;10(6):1183. doi: 10.3390/microorganisms10061183 (PMC9230084; doi:10.3390/microorganisms10061183)
Supplement: Supplementary file 1 [file microorganisms-10-01183-s001.zip › Figure S1.pdf]

PVX_050690

```
      M  P  E  S  V  T  L  N  E  I  F  A  R  T  N  I  N  E  V  D  K
1.    GGTACCATGCCAGAAAGTGTAACTTTAAATGAAATATTTGCTAGAACAAATATTAATGAAGTAGATAAA
      K  R  Y  D  S  A  F  S  E  L  L  R  H  I  N  H  G  H  V  F  F  N  Y
70.   AAAAGATATGATAGTGCTTTTAGTGAATTATTAAGACATATTAATCATGGACATGTATTTTTTAATTAT
      N  K  E  G  C  W  Y  I  S  Y  I  L  H  K  E  V  E  E  I  L  G  Q  Y
139.  AATAAAGAAGGATGTTGGTATATTAGTTATATATTACATAAAGAAGTTGAAGAAATATTAGGACAATAT
      Y  N  S  Y  A  Y  D  L  L  K  Q  F  V  F  K  F  N  E  N  R  S  I  R
208.  TATAATAGTTATGCATATGATTTATTAAAACAATTTGTTTTTAAATTTAATGAAAATAGAAGTATAAGA
      S  D  I  C  K  K  D  Y  V  Y  I  N  S  D  T  Y  K  I  M  N  G  L  Y
277.  TCAGATATATGTAAAAAAGATTATGTATATATTAATAGTGATACTTATAAAATTATGAATGGATTATAT
      N  L  Y  D  E  Y  T  K  Y  K  P  I  H  D  N  T  I  P  K  N  C  C  F
346.  AATTTATATGATGAATATACAAAATATAAACCTATTCATGATAATACAATACCAAAAAATTGTTGTTTT
      L  Y  E  N  I  K  H  F  D  T  E  I  R  K  T  I  L  K  Y  K  I  Y  E
415.  TTATATGAAAATATTAAACATTTTGATACAGAAATTAGAAAAACAATATTAAAATATAAAATATATGAA
      C  R  E  Y  P  Y  E  V  G  K  L  N  L  Y  T  P  P  E  P  E  K  T  I
484.  TGTAGAGAATATCCATATGAAGTTGGAAAATTAAATTTATATACACCACCAGAACCAGAAAAAACAATT
      A  P  V  P  V  E  Q  Q  Q  A  P  N  V  L  Q  N  S  H  H  E  V  T  H
553.  GCACCAGTACCAGTAGAACAACAACAAGCACCAAATGTATTACAAAATTCACATCATGAAGTAACACAT
      H  P  V  T  A  V  D  K  V  R  K  V  E  A  E  Q  E  Q  G  T  A  H  D
622.  CATCCAGTAACAGCAGTTGATAAAGTTAGAAAAGTAGAAGCTGAACAAGAACAAGGTACTGCACATGAT
      Q  E  I  A  R  D  Y  V  N  S  P  E  H  G  R  V  F  K  P  E  G  V  H
691.  CAAGAAATAGCAAGAGATTATGTTAATAGTCCAGAACATGGAAGAGTTTTTAAACCTGAAGGTGTTCAT
      N  A  E  R  S  R  E  H  G  I  S  H  G  Y  R  S  T  Y  N  H  R  G  E
760.  AATGCAGAAAGATCAAGAGAACATGGTATAAGTCATGGATATAGAAGTACTTATAATCATAGAGGTGAA
      E  K  T  F  R  P  G  L  S  P  Y  P  Q  A  F  A  T  H  D  S  S  V  K
829.  GAAAAAACTTTTAGACCAGGATTAAGTCCATATCCACAAGCTTTTGCTACACATGATAGTAGTGTAAAA
      Q  E  L  T  D  V  S  S  F  S  Q  K  S  E  P  Q  K  E  D  E  G  F  M
898.  CAAGAATTAACAGATGTATCAAGTTTTAGTCAAAAAAGTGAACCACAAAAAGAAGATGAAGGTTTTATG
      A  N  M  R  N  T  I  T  G  V  L  G  E  V  D  P  V  P  V  V  G  V  S
967.  GCAAATATGAGAAATACAATTACTGGTGTATTAGGTGAAGTTGATCCAGTTCCAGTAGTTGGAGTTAGT
      G  G  M  G  A  L  F  L  L  F  R  Y  T  P  V  G  A  F  F  R  G  G  R
1036. GGTGGAATGGGAGCATTATTTTTATTATTTAGATATACACCTGTTGGAGCATTTTTTAGAGGTGGAAGA
      G  R  V  H  R  I  P  R  S  F  N  G  P  F  P  G  G  F  P  G  F  E  E
1105. GGAAGAGTTCATAGAATACCTAGAAGTTTTAATGGACCATTTCCTGGTGGATTTCCAGGATTTGAAGAA
      Y  E  S  G  Y  I  G  Y  G  P  M  N  P  L  A  E
1174. TATGAATCAGGATATATTGGATATGGTCCAATGAATCCATTAGCAGAACCTAGG
```

PVX_ 060690

```
      M  G  S  I  T  I  N  I  Y  D  K  D  Q  T  L  D  S  Q  E  C  L
1.    GGTACCATGGGAAGTATAACTATAAATATATATGATAAAGATCAAACATTAGATAGTCAAGAATGTTTA
      E  R  Y  F  E  I  L  E  N  I  Q  G  S  I  A  E  F  E  R  K  E  Y  A
70.   GAAAGATATTTTGAAATATTAGAAAATATTCAAGGATCAATAGCAGAATTTGAAAGAAAAGAATATGCA
      D  F  N  K  D  W  E  E  L  I  K  Y  I  N  E  Q  K  K  K  L  K  I  C
139.  GATTTTAATAAAGATTGGGAAGAATTAATTAAATATATTAATGAACAAAAAAAAAAATTAAAAATTTGT
      Y  D  R  N  L  L  E  I  H  L  N  S  D  A  D  I  S  G  F  Y  R  K  C
208.  TATGATAGAAATTTATTAGAAATTCATTTAAATAGTGATGCAGATATATCAGGATTTTATAGAAAATGT
      N  A  N  P  K  C  L  N  Y  I  S  P  Q  D  K  K  L  T  E  L  K  D  D
277.  AATGCAAATCCAAAATGTTTAAATTATATTAGTCCACAAGATAAAAAATTAACAGAATTAAAAGATGAT
      T  E  T  S  C  E  G  R  N  C  G  K  K  V  K  A  A  K  G  K  T  G  V
346.  ACAGAAACAAGTTGTGAAGGTAGAAATTGTGGAAAAAAAGTAAAAGCAGCAAAAGGTAAAACAGGTGTA
      K  T  P  Q  L  N  E  K  G  S  V  M  K  T  L  E  T  Q  K  S  Q  G  Q
415.  AAAACACCACAATTAAATGAAAAAGGATCAGTAATGAAAACTTTAGAAACACAAAAAAGTCAAGGACAA
      G  E  N  H  V  D  G  K  E  P  R  Q  E  Q  V  I  S  Q  D  H  P  H  V
484.  GGTGAAAATCATGTAGATGGAAAAGAACCTAGACAAGAACAAGTTATAAGTCAAGATCATCCACATGTT
      I  S  L  R  P  S  V  E  P  K  S  D  G  S  E  P  K  L  D  H  H  S  N
553.  ATAAGTTTAAGACCAAGTGTAGAACCTAAAAGTGATGGAAGTGAACCTAAATTAGATCATCATAGTAAT
      P  H  I  P  I  D  N  P  T  Q  R  L  G  I  A  P  Q  P  T  H  R  A  L
622.  CCACATATACCAATAGATAATCCAACACAAAGATTAGGAATAGCACCACAACCTACACATAGAGCATTA
      D  S  N  P  S  D  S  S  L  Q  N  S  S  E  G  D  S  P  S  I  G  T  S
691.  GATTCAAATCCAAGTGATAGTAGTTTACAAAATTCAAGTGAAGGTGATAGTCCAAGTATAGGAACAAGT
      Q  G  K  T  P  E  T  V  A  S  Q  R  D  T  R  N  S  Q  T  L  N  D  N
760.  CAAGGTAAAACTCCAGAAACAGTAGCAAGTCAAAGAGATACAAGAAATAGTCAAACTTTAAATGATAAT
      A  H  G  K  Q  A  V  G  S  Q  T  Y  E  E  H  T  S  V  D  K  D  A  V
829.  GCACATGGAAAACAAGCAGTTGGAAGTCAAACATATGAAGAACATACTTCAGTAGATAAAGATGCAGTA
      V  A  S  A  T  E  N  R  D  V  G  T  S  I  S  G  N  P  G  H  R  N  P
898.  GTAGCTAGTGCAACAGAAAATAGAGATGTTGGTACAAGTATAAGTGGAAATCCAGGACATAGAAATCCA
      P  P  I  V  T  D  G  A  V  D  G  S  I  S  L  P  S  V  G  A  T  G  I
967.  CCACCAATAGTAACAGATGGTGCAGTAGATGGTAGTATATCATTACCAAGTGTTGGAGCAACTGGTATA
      K  G  T  T  E  L  S  G  H  A  I  Y  S  V  E  C  T  T  G  S  A  L  P
1036. AAAGGTACTACAGAATTATCAGGACATGCAATATATTCAGTAGAATGTACAACAGGATCAGCATTACCT
      G  E  V  D  T  T  K  A  A  G  N  S  P  T  D  S  K  A  A  D  E  V  L
1105. GGTGAAGTAGATACAACAAAAGCAGCTGGAAATAGTCCAACAGATAGTAAAGCAGCAGATGAAGTATTA
      G  V  E  A  K  Q  D  K  V  P  C  S  E  N  P  C  N  N  D  S  H  Q  V
1174. GGTGTAGAAGCAAAACAAGATAAAGTACCATGTAGTGAAAATCCTTGTAATAATGATAGTCATCAAGTT
      F  S  V  G  T  S  C  S  E  A  L  S  N  C  Q  K  I  Q  G  N  R  D  S
1243. TTTAGTGTAGGTACAAGTTGTAGTGAAGCTTTAAGTAATTGTCAAAAAATTCAAGGTAATAGAGATAGT
      S  A  S  V  S  N  S  A  E  L  Q  M  D  N  S  T  Q  S  K  P  H  T  E
1312. AGTGCAAGTGTTAGTAATAGTGCAGAATTACAAATGGATAATAGTACACAAAGTAAACCACATACAGAA
      Q  E  A  S  D  Q  G  N  E  L  Q  K  E  K  T  R  T  D  R  E  S  N  Q
1381. CAAGAAGCTTCAGATCAAGGTAATGAATTACAAAAAGAAAAAACAAGAACAGATAGAGAAAGTAATCAA
      E  V  H  S  G  K  E  H  R  S  N  M  Q  Q  S  Q  T  Q  G  G  H  E  G
1450. GAAGTACATTCTGGAAAAGAACATAGAAGTAATATGCAACAATCACAAACACAAGGTGGACATGAAGGA
      S  T  E  G  L  H  H  V  N  H  E  T  S  E  G  G  K  Q  Y  L  Q  D  K
1519. TCAACTGAAGGATTACATCATGTAAATCATGAAACATCAGAAGGTGGAAAACAATATTTACAAGATAAA
      H  P  Q  P  T  T  C  V  P  T  S  T  I  N  D  G  E  T  L  V  S  S  Q
1588. CATCCTCAACCTACAACATGTGTTCCAACAAGTACTATAAATGATGGTGAAACATTAGTATCTAGTCAA
      S  N  R  S  G  A  G  A  T  G  E  A  V  T  T  G  S  G  N  V  L  D  I
1657. AGTAATAGAAGTGGTGCTGGTGCAACTGGTGAAGCAGTAACAACAGGTTCTGGAAATGTATTAGATATT
      F  N  R  L  F  S  N  V  P  Y  V  D  K  E  Y  I  M  M  A  L  V  P  L
1726. TTTAATAGATTATTTTCAAATGTACCATATGTCGACAAAGAATATATTATGATGGCATTAGTACCATTA
      A  I  I  L  L  L  T  F  L  I  K  F  T  P  L  G  T  F  F  T  K  K  K
1795. GCTATTATATTGTTATTAACCTTTTTAATAAAATTTACACCATTAGGAACATTTTTTACAAAAAAAAAA
      K  K  E  Q  K  K  M  N  E  K  L  Q  R  V  L  S  E  Y  P  A  Q  M  N
1864. AAGAAAGAACAAAAAAAAATGAATGAAAAATTACAAAGAGTATTATCAGAATATCCAGCACAAATGAAT
      E  R  N  I  P  F  S  Y  S  P  F  A  Y  S  T  Q
1933. GAAAGAAATATTCCTTTTAGTTATAGTCCATTTGCTTATTCAACACAACCTAGG
```

PVX_093715

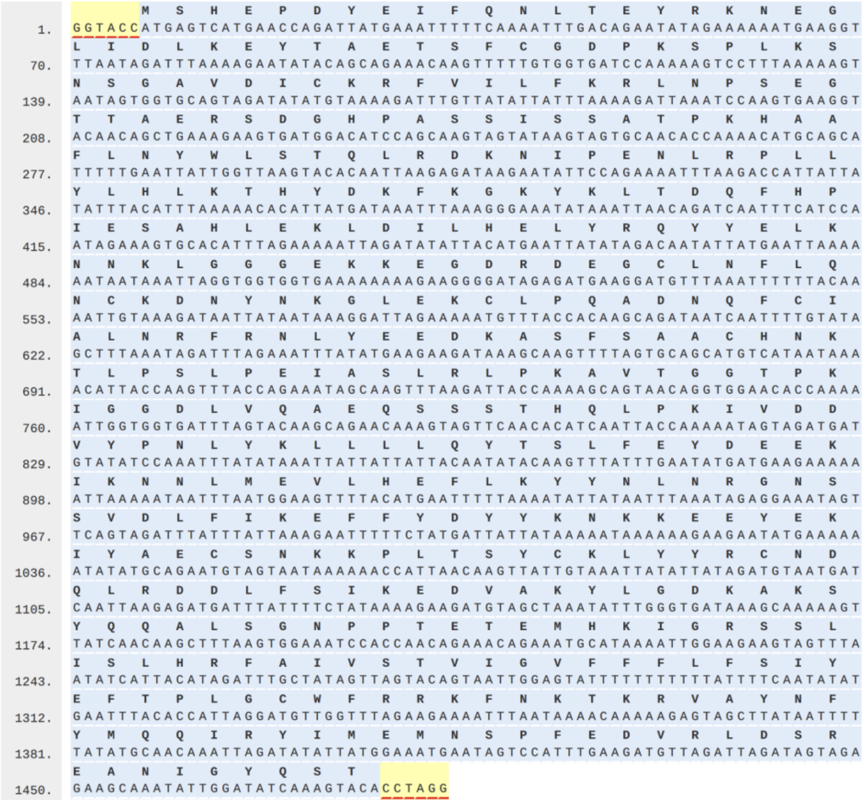

PVX_101560

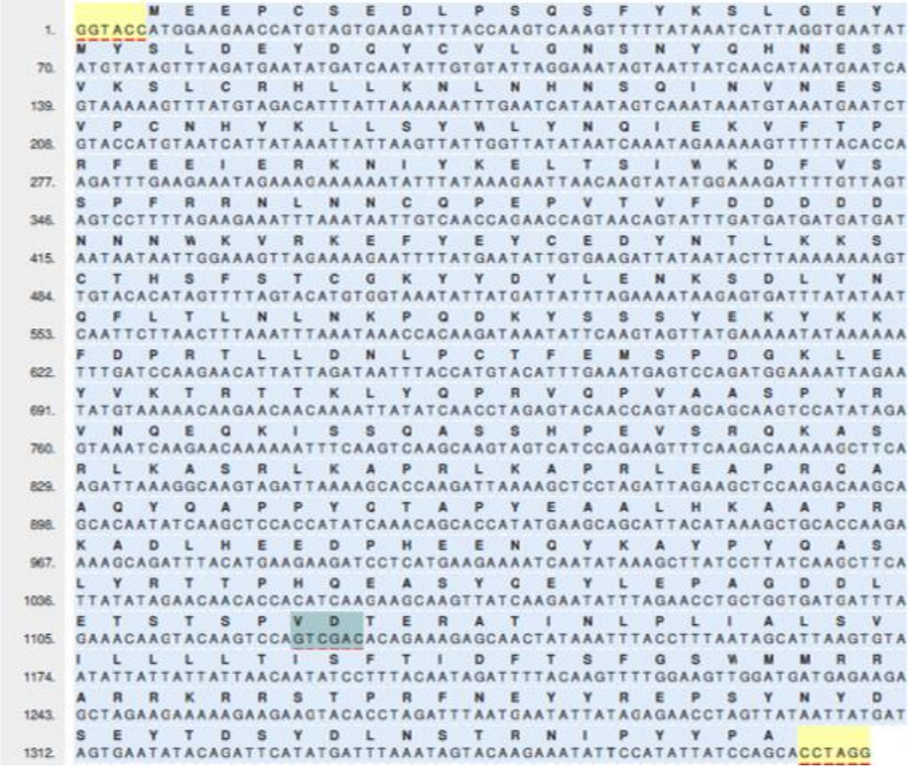

PVX_113230

```
            M  E  K  D  P  K  Y  K  D  L  P  S  Q  I  Y  Y  D  K  L  N  E
1.    GGTACCATGGAAAAAGATCCTAAATATAAAGATTTACCAAGTCAAATATATTATGATAAATTAAATGAA
      D  I  I  E  E  E  D  D  E  H  E  E  N  E  E  S  Q  E  N  V  Y  W  E
70.   GATATTATAGAAGAAGAAGATGATGAACATGAAGAAAATGAAGAATCACAAGAAAATGTATATTGGGAA
      A  I  E  G  S  Y  E  Q  T  P  W  V  R  D  V  F  F  K  L  E  R  N  L
139.  GCAATAGAAGGATCATATGAACAAACACCATGGGTTAGAGATGTATTTTTTAAATTAGAAAGAAATTTA
      T  E  I  N  E  S  R  G  E  D  S  L  S  K  K  H  C  Y  D  L  N  Y  W
208.  ACAGAAATAAATGAATCAAGAGGTGAAGATAGTTTAAGTAAAAAACATTGTTATGATTTAAATTATTGG
      L  Y  E  Q  V  Y  E  N  L  N  N  N  E  N  D  E  N  F  F  K  I  I  D
277.  TTATATGAACAAGTATATGAAAATTTAAATAATAATGAAAATGATGAAAATTTTTTTAAAATTATAGAT
      G  L  Q  N  A  W  T  N  I  N  N  D  K  F  P  N  A  D  N  I  C  H  P
346.  GGATTACAAAATGCATGGACAAATATTAATAATGATAAATTTCCAAATGCAGATAATATTTGTCATCCA
      D  K  T  L  V  D  M  K  Y  L  K  D  V  K  H  L  F  D  F  I  E  D  F
415.  GATAAAACATTAGTAGATATGAAATATTTAAAAGATGTAAAACATTTATTTGATTTTATTGAAGATTTT
      S  T  I  K  T  A  A  I  K  D  T  N  N  A  C  Q  K  Y  I  D  Y  L  K
484.  TCAACTATAAAAACAGCAGCTATAAAAGATACAAATAATGCATGTCAAAAATATATTGATTATTTAAAG
      L  K  V  P  L  Y  Y  E  W  N  D  V  C  T  M  E  E  E  N  I  C  T  K
553.  TTAAAAGTACCATTATATTATGAATGGAATGATGTATGTACAATGGAAGAAGAAAATATTTGTACTAAA
      Y  I  D  D  Y  P  K  Y  N  P  K  N  V  L  E  N  L  S  V  V  S  L  A
622.  TATATTGATGATTATCCTAAATATAATCCAAAAAATGTATTAGAAAATTTAAGTGTAGTTAGTTTAGCA
      L  A  S  I  F  N  D  C  Y  Q  N  I  I  N  L  F  T  E  A  E  K  I  E
691.  TTAGCAAGTATTTTCAATGATTGTTATCAAAATATTATTAATTTGTTTACAGAAGCAGAAAAAATTGAA
      P  R  T  V  L  K  H  R  D  I  T  G  P  S  E  S  N  V  V  K  I  G  G
760.  CCTAGAACAGTTTTAAAACATAGAGATATTACAGGACCAAGTGAAAGTAATGTAGTAAAAATTGGAGGT
      R  A  L  A  E  A  I  S  D  T  S  Q  S  G  N  M  L  I  G  I  N  A  L
829.  AGAGCATTAGCTGAAGCTATAAGTGATACAAGTCAAAGTGGAAATATGTTAATAGGTATAAATGCATTA
      A  T  S  L  F  V  D  S  V  V  K  R  F  N  S  Y  V  F  S  L  V  A  P
898.  GCTACAAGTTTATTTGTCGACAGTGTTGTAAAAAGATTTAATAGTTATGTTTTTAGTTTAGTAGCACCA
      V  G  L  S  L  L  G  L  L  L  F  L  Y  V  L  Y  K  F  T  P  I  G  K
967.  GTAGGATTAAGTTTATTAGGATTATTATTATTTTTATATGTATTATATAAATTTACACCAATAGGAAAA
      S  I  S  R  T  H  K  R  V  K  N  K  F  V  R  N  K  R  D  D  F  D  D
1036. AGTATAAGTAGAACACATAAAAGAGTAAAAAATAAATTTGTTAGAAATAAAAGAGATGATTTTGATGAT
      D  E  D  D  D  D  D  D  S  D  N  S  S  D  L  K  S  S  S  S  M  E  S
1105. GATGAAGATGATGATGATGATGATAGTGATAATAGTAGTGATTTAAAAAGTAGTAGTTCAATGGAATCT
      L  L  S  N  S
1174. TTATTAAGTAATAGTCCTAGG
```

PVX_115475

```
            M  E  E  E  D  L  D  S  K  Y  E  N  V  P  S  Q  I  F  Y  K  E
1.    GGTACCATGGAAGAAGAGGATTTGGACTCGAAATATGAGAACGTGCCCTCACAGATATTTTACAAAGAA
      L  N  A  E  L  K  D  R  V  N  T  Q  W  E  K  L  K  D  L  I  E  E  Q
70.   CTGAATGCTGAATTGAAGGACAGGGTTAATACACAATGGGAAAAGTTAAAAGATTTAATAGAAGAACAA
      P  L  L  K  D  V  C  D  K  L  E  K  N  L  K  S  L  N  A  N  P  Q  S
139.  CCTTTACTTAAAGATGTTTGCGACAAACTTGAAAAAAATTTAAAATCCTTAAACGCTAATCCTCAGTCA
      E  M  L  S  K  K  H  C  Y  D  I  N  Y  W  L  F  D  N  V  H  N  K  L
208.  GAGATGTTAAGTAAGAAGCATTGTTATGATATAAATTACTGGTTATTTGATAATGTACATAACAAGCTT
      N  I  K  E  E  D  P  L  F  Y  N  I  I  D  S  V  H  S  V  W  R  D  I
277.  AATATTAAAGAGGAGGACCCTCTTTTTTATAACATTATCGATAGTGTCCATAGTGTATGGAGAGATATA
      N  E  S  L  P  D  K  T  H  I  C  K  P  D  S  T  L  M  D  M  P  V  L
346.  AATGAGAGTTTGCCAGATAAGACTCATATATGTAAACCAGATTCAACGTTAATGGACATGCCTGTTTTG
      K  E  F  K  H  L  F  D  F  I  E  N  F  A  F  F  K  A  E  A  F  K  D
415.  AAGGAATTTAAGCATTTGTTTGATTTTATTGAAAACTTTGCGTTTTTTAAAGCAGAAGCTTTTAAGGAT
      T  P  K  A  C  T  K  Y  F  K  Y  L  E  R  S  V  Q  I  Y  Y  A  R  E
484.  ACTCCCAAGGCATGCACGAAATATTTTAAATACCTTGAACGTAGTGTCCAAATATATTATGCAAGGGAG
      I  F  C  T  N  P  E  S  N  M  C  N  R  Y  I  D  N  Y  K  S  Y  N  P
553.  ATCTTTTGTACTAACCCAGAAAGTAATATGTGCAATAGATATATTGATAACTACAAATCTTATAATCCA
      K  N  V  R  E  E  L  N  V  S  K  L  I  M  G  L  V  W  Y  Q  C  Y  R
622.  AAAAATGTTAGAGAAGAATTAAACGTGTCTAAACTTATTATGGGTTTGGTTTGGTATCAATGTTACAGA
      D  V  V  S  L  F  R  A  V  K  K  Q  P  Y  R  L  E  V  K  F  R  T  V
691.  GATGTTGTAAGTTTATTTAGAGCTGTAAAAAAACAGCCTTACCGTTTAGAAGTAAAATTCAGAACTGTC
      Q  G  D  V  G  T  T  V  V  V  P  D  A  I  K  E  F  F  R  A  V  Y  S
760.  CAAGGGGATGTTGGAACAACTGTAGTGGTTCCTGATGCGATAAAAGAGTTCTTTCGTGCAGTTTATTCT
      V  L  K  H  I  Y  G  I  V  Y  F  L  I  F  L  P  V  L  F  I  L  G  P
829.  GTCTTGAAACATATATATGGTATTGTGTATTTTTTGATATTTCTGCCCGTTCTTTTTATTTTGGGTCCA
      Y  V  F  F  L  L  I  Y  K  F  T  P  L  G  R  Q  M  L  R  A  R  A  L
898.  TATGTCTTTTTTTTATTAATATACAAGTTTACTCCACTCGGAAGACAAATGTTACGTGCCCGAGCACTA
      L  K  K  K  L  K  T  N  I  S  Y  E  D  I  I  L  L  N  G  S  S  E  S
967.  CTAAAGAAAAAATTGAAAACCAACATTAGTTATGAGGACATCATATTGCTAAATGGAAGTTCCGAATCT
      I  F  S  G  S  S  S  D  S  S  Y  I  V  G  Y  Q  A  S  S  Q  S  S  S
1036. ATTTTTAGTGGTAGTTCATCGGATAGCTCATATATTGTAGGATACCAAGCTTCTTCCCAGAGTAGTTCA
      G
1105. GGACCTAGG
```

PVX_068690

GGTACC

ATGAGTTTTAGAAATAGATGGAGTTATAATTATTGGCAACAATATGAAGGAGCAAGTTGTTATAATAATTATAGT
ACATATAAAAGAGAAATTGAAGAAAAAATAGATAATTTATATAGAATAACAAATGGAAATTTTTATACACAATGG
CATCAATTAAATGAATATATTAAAAAGAAAAATAATGAAATTAAAAATTGTGATAGAAATAAAAGTACATTAGAT
TTATTAAAAGATGATAATATTAAAAGATTTTCAACATTATGTAGTAATAGATTATCATGTAGAACAAAACCAAGT
TCATTAGTTAATAATCATGATAGTTTACAACCAGGAAAAAATGCAACATGTAAAGGAGATTCATGTCCACAAAAG
AAAACAAGAACAAAAAGTCCAGTTGCAAAATTACAATCATTACCACATACAGGTAGTTCAAATGCTAAAAGTTCA
TTAAATCCAAAAACAATAAATCCAGTACAAGAACATCATGAAAGAAAAGGAAGTGAACAACCATCAATTAGTTCA
CAAGCTCATCAAGAACAAAAACATAGAGGTAGTTCAGTTCAAACAGAAGGTGGAAGTCCAGAATCATTAAAAGTA
CATAATACAGCAAAACCAGAATTAGAACAAAGTTCAGCACAAAGTGCTTCAATACCAGCTCCAGTTACAACAACA
GAAGTAATAACACATCAAATTAGTCATTCACCAGGAAGTAAAGCATCAGAAGAAAGTGATGCTAGTTCACCAGTT
CAAGAAATTGATTTAAAAGGTAGTAATCATCAATCAGGAACAAGTGCAGGTCAAACAAGTGATGATAATTTACCA
AATTTACAATCAATAAATGGAATTACAGATGCTAATCAAGATCCAAATAATCAAATATTTAGAGTTGAAATAGAA
TATGTTAGTGATGAAAATCCACCAGGAACATCAACAGGTGATGTAAGTTCAAGTTCAACAGATACAGCATGTGCT
GATACAGATAAAACAAATATACATCATGCAACAACATGTAATAAAACTTATAGTGGAATACCAACAAATCCAGAA
ACATCAGGTGATGAAGCTACACCAGATGAATTAGTTAGAGGAGAAGGTGCAGTTGTAGCTGATGTTAGAAGTGAA
ACAAATGGAACAGAAGATGCAGAAAATGGTCCATCAGTTAGAGAAAAAACAGATGTTATTGTATTTAGTGATAAT
GGAGTAGATCATAAATCATTTTGTTTAGAAGATGCTGAAAATCCAACACATGAAAATGGAATACCATGTATTGCA
GAAAAAGGTACAGAAATTGATGCTAATAATGGAAATATTTTAGGTACATTAAGAGAATTTTTCTATGAAATACAA
AATAATCCACATATAATTAAAACAAGTATACCAATTGGTATTATATTTTTGTTAACATTATTATTTAAATATACA
CCATTATGGAGAATATTAACTAAAAAGAAAAAGAAAGAACCAGTTGATATGAATGAAGAATTACATTCAGTATTA
CAAGAACCATTAATTATGGATGATGAAAGATCAATACCTTTTAGTTATGGAGCATTTGAATATAGTACATTTGAT
GAAAATACTTAT

CCTAGG

PVX_077695

GGTACC

ATGGAAAAAGCATTATTAAGTTTACAATCAAAAAGAAATGCAGTTATAGCAATAGAATATAAAAAATTAGATGAT
TTAGATATTAAAGATTATTCAGATAATTATTGTGAAAAAGATTTAGGTAGTCCAAAGAAAGAAGATAAAGAATTA
TGTAATAAAGTATCAAAACATTTAAAAAGATTAAGTGGTATATCAAATAATGATGATAGAAAACATGGATGTTTT
TATTTTCAATATTGGTTTTATGATCAAATAAGTAAAAAATATTCAGCTGATGATAAAATTAATAATAAACAAGTA
TCAGATAAATTGTTTGATTTAGTTGCAACAACAATTTTAAAAAGTCCAAATTTAGAACCATGTAGATGTTATGAA
AGTGGTACACCATCAATATGGAAAGAAGAAAAAGATTTACATGATTATTTTAAAAATTATAAAGATATAAATTGT
ACAAATTCAGATAAAACAACATGTGAAAAATATGTTAGATATGTAACATATATAGATAAATTGTTTCAAAATAAA
GAAGATACATGTTGTTATGATGAAGATGTAGAAAGTTTTTGTGAACATTATATAAATTGTAATAATAAATATAGA
CCAGATGGATTATTAACAAAATTACAAACAGAATTAAAAGCATTAGATGCAAAAGTTAAAGAAGTACCAAAAGCT
GTTGGAGGTGAAGATGCTCCAGGTGCAGTTGTAGAAAATAAAGCAGCTGGAAGTGATGGTCCAGGATCAGAAAAG
AAAGCACCAGGTATAGCTGGAGCAGAAGAAGCTAAACAATTACCTGCAAAACCAGTAGCAGCTAAACCAGTTGGT
GAAGATCCACCAGCAGCTGAACCATTAGCAGCTAAACCAGCAGCTGCAAAACCAGTAGCAGCAGAAAGTGGAGGA
TTACCACCAGAAGTAGCTAAACCACCAGCAACAGTTAGTGGAGGTACAGAAAGTGGAGGAACAGAAAGTGGAGGT
GGAAAACCAGCAGAAGTAAAACCAGAAGTTGTAAAACAAGAAACAGTTGGACCAGCTGAACAAGAAGCACCACCA
CCACCACCAGCTCCAAGAGAAACAGTTCAACAACAACCAGCACCATTAGAACCAAAAGAAAGTTTAGATGAAGAA
GAAGTTGCTGAAGTAACAGAAGATGGTGTTGATGAAGTAGATGAAGTTGCAGAAGAAGGTGAACCAGGACAATTA
GAAGGAGAAGCAGCAGTTATATTACAAGATACAGCTACATTTAGTGCACCATCATTAGAAGATTCAGGTGCAGCA
GTTCATGCTGCACCATATTATACACCAGAATTAGCTGGTAATGGAGCACCATTAACAAATAGTGAAGCACCATCA
ACATTAAGAACAACACATGAAGAATTAGATAGTAATTTCTTTAGAAATGTAATTATGGCTATAGCAGTTTTAGGA
ACAATATGTTTCTTATTTTATTATAATAGAAGTTCAAGATTAGAACCAAATAGTAGAAAGAAAAAGAAAAAGAAA
GGAAAAATATTCGAACATAATTATTATGAAGAATATGAAAAAGAATTAGAAATGTATGGAAGTGAAGAAACATTT
ATAGATTCAGAAACAGATAGATTATATTTAAATTATCATCCAGATCAAGATAGTTATTAT

CCTAGG

PVX_081850

GGTACC

ATGGCACAAAATATAGTAGATCAAGCATTATCAGTATTAAAAGAAGGAAATTATACAGTAAGAGCAGCAACTAAA
TTAATATTCTTTTATGATATTTTTGATACAGATTTTGATAATTTTTATAAAATTAAATTGTGTAATGGATGTGAT
AAAAATAAATATTTATTTGATAGTTTAAGAAAAACAATAAAACAAATAATTGAAGAATGGGAAAGTTTATTAGAT
TTCTTTAGATGTGAAACAGCAGGAGATGAAAATACATGTTGTTTAAATTTTATATATTGGATTTATGGTAAAATA
AAAGATAGTAATTTAAATGTTGATTATATAAAAGAAATTTATAATAATTTAGATGAATTTGTAAAACAAAATTGT
TTTGGTTATGATGCAGAAAAACCAGAAAAATTTTTCAAAAAATATGTTAAAGCCTATGATAAGAAAGTTTTAAAA
AGAAAGAAAGAATTATATGATTTTGTTCAATTTTATGAAAGAGTAAAAAGTAAATTGGATGCTGTTCATTCAAAA
AGAAAAGTAATGTGTGATTATATTAGTTATATATTTAAAATGTATGAAGAAATGAGAAGAGATTGTAGTTCAATT
AAATCAGAATGGTATGAAGATGAAATGAAAAATTTTATTGAAATGTTAAAGAAAAATAATGAATTAACATTATTA
GAAAGTAAATGTGGAGATATTACAAAAGGTTTAATTTTAGATGATAATATTGAAGCATTATGTCCATCAAATAAT
GAAAGTGCAGCTAGTAAGAAAGGACAAATATTAAAAAGTCCACAAGAAGAAGAAAAATTTAATTTATCAGCTGTT
CCAAAAGTAAATATGCAAAGTGATGTTTCAAATCAATTACCTAGTAAGAAAATATGTGAAGAAATGGATAAAAAT
AATAATCCAGATTTAAATATTACAGATTGTAATGTATTTAAAGATCAAATTGGTAAATCATTATGTGCACATACA
ATTAAAAATTTTAAGAAAATAAATAAAATTGAAGATAGAGAACAACATAATGATTATTGTTTACATTTTATGTAT
TGGTTATATGGAAAAATTGGTGAAAATTATATAAATAAAAGTTCAAATATTTTAGAAGATCCAGAAATAAGTAAA
TTTGTTCATTTAATTGTATCATTAAATAATAAAAGTTCAAAATATTATTGTTATTATAATAGTGAAAATAATTTA
GAAGAATGGAAAGAAATAATTGATTTAATAGATTATTTTAAAATACATAATAATATTGATAATACAAAATCATGT
GATGATAATGCATGTGCTAAAAATTGTGAATATTTTAGTAATATATCAAATTTATATAAAAGACATAAAAGACAA
TGTTGTACATATTTTTATTATGGAGATCATTTTAATAATTGTACACATTATTTTAAATGTGATGATAAATATAGT
CCAGATTCAATGTTAACAAGATTAAAATGTCCAGTTAATGAAAATGAAAAAGATATTAATGAAGAAGATGAAGTT
GTAGGTTTTGATCAAAATGTAATATTACAAAGTTTAGATTCATTAAAAAGATTTAATCAAAGTAAAAATATATGT
GATGGAATTATGTGTGATACATTTAGTAAATTTGCATTTTTAGCTTTTTCATTTTTAGGTGTTTTAGTAACATTT
TTCGTATTTTATAAATTTACACCATTAGGAATATGGTTAAATAGAAAAGGTTTTAAGAAAAATAAAGCTAGATAT
GATTATTATGAAGAATCACCACAACAATTATTAGAAAATAATAGTAAACCTGTTCAAAGAAATATGCAAAATGGA
AGAATTAGAATAGCTTATCAATCA

CCTAGG

PVX_096925

GGTACC

ATGAAAATAATAGATCATTTAATAGAAAATATAAGAATAGATGCAATACCTACTTGTCCTAGAAATATATTTAGT
GGAAAATGTGATCATCCAAGTTTTCATTATTATTTTGTATTAGCACAAAGATATTTAAGTGATTTAATAAAGAAA
GAAAGTACAATAAATGTTGCACATGCTTGTAAATTTTTAAATTATTGGATATATAAATTTGTATGGAATGGAGTT
ATGTATAATAAAAATATGAGTACATTTTATGAAGAATTAAAAGATGGTTCAGATAATGAAGATATTTGTAAAAAT
TATATGGAAGATATAGATGAAAATACTTATGAATATATTTTAAAATTGATAGATTTATATACAAATTTAAGTAAT
TTATCAAAACCACATAATGGAAATAAATGTCCAACAATTAAAACATGTTTTGATAGTTATATGCAATGTAAAGAT
ACATGTAAAGGTGATGAAAATAAAAATTTTTGTAATGAATTAGAAAATTTTAGAAAAAGATATAATGTAGCAATG
AAAAGTGTAAATAATTGTGTTGATGAACATAAATATTTACCAAGTTTTCAAGATTCACCAATTGTTCCAGTATCA
GTTATACCAATAATTATAACAAGTGTTATTTCATTAATTTTAATAATTAGTTGTAAATTTACACCATTAGGAAAA
TGGATATGTCCAAGATCAAAAAGAGCTAAAAATGGAACAAAGAAAATAAATCATGAAATGAATAAATCACAACAT
ACTCCTGGTTATCAAGATATGTCTTATTTAGTTAGTTATCAAAGTCCAAATCCTTCT

CCTAGG

PVX_097525

GGTACC

ATGGCAGCAAGTACAGGAAATAGTTGGGATGAAGCATTATTACATTTACCAGCATATCAAAAATATAAAGAATTT

GATTCAGTAGATATATCAAAAGAAACAACAAGTCAATGTAATAATTTAGGATCAAAAGAAGAAAGTGATAAAACA

TTATGTAAGAAAATAGCACAAAATTTAAGAAAATTATCAACATTACAAGGAGATGAATTAAAAAATGGTTGTTAT

TATTTTCAACATTGGTTTTATGAACAAATAGCTAAAACATATTATGATGGAAAAAATAAAAATAATAAATATCAT

GTAGGTGAAACATTATTTGATATAATTGCATTATTTATTTCAACATATCCAAAATTAGAACCATGTAGATGTTAT

GTTAGTGGAAAACCAGAATATTGGGAAGAAGAAAAATATTTACATGATTATTTTAAAAATTATCAAGATATAAAA

TGTAGTAATAGTTCAAAAGATAGATGTGAAAAATATATACAATATGTAACATATATTAATAGATTATTTCCAGCT

AAAGAAGATACATGTTGTGATGAAGGAGAATTAATTGAAGATTTTTGTAAACCATATTTTAATTGTGAAAATAAA

TTTTCACCAGAAAAATTGTTAACACAATTAAAAACAGAATTACAAAGTTTAGGAACAAAAGCTGAAGCACCAAGA

GAAGGTGGAACAGTTGGAGGTGTTGTAGATGCTAAAGCAAAACCAGGTGCAGCTGAATCAGAAGGAGCAGAAAGT

GGTATACCAAAACCAGCAGCTGCAAAACCAGCTCCAGCAAAACCAGTAGCTACATCACCAGCACCAAGTGAAAGA

GCACCAGCAAAACCAGTTGCTGCAAAACCAGCTGCAACAAAACCAGCTACAACAAAACCAGCAAAAGAAGAATCA

GAAGGAGAAGATCCAGCTGGTGCAAAACCAGTAGCAGCTAAACCAGCTCCAGGAGAAGCTGTTCCAGCTAAACCT

GTAGCTGCAAAACCAGTTGCAACAGAATCAGCTGCACCAGAAAGAGCACCAGAAGCACCAGAACCAAAAAGAAGT

CAACCAGAAATACCAGCTAGAGAAGTACCAGAACAAGCTGTTGTAGAAACAGCAGAAACATTACAACATGAAGAA

TCATTAAGTCCACCACCAATGGAAGGTTCAGAACCAAGTGTTCAAGAAGTAAGTTCATATAATTCAGAATTAGCT

AGTAATGGAGTTCCATTAACAATAGCAGATAGTCCAAATACATTAGGTACAACAAATGAAGAATTAGATTCAAAT

TTCTTTAGTAATATAATAATGGCTGTTGCAGTATTAGGAACAATATTTTTCTTATTTTATTATAATAGATCAGCA

AGATTAGAAAGTAGTAGTAGAAAGAAAAAACAAAAGAAAGGAAAAATATTCGAACATAATTATTATGAAGAATAT

GAAAAAGAATTACCAATGTATGATAGTGAAGAAACATTTGTAGATTCAGAAATGGATAGATTATATTTAAATTAT

CATCCAGATCAAGATAGTTATTAT

CCTAGG

PVX_107235

GGTACC

ATGGCATTAAGTTCTGAAAAGAATTGGGTTGAAGTATTACAAAATTTACCTAGTTATAAAGAATATGAAAAATTA

GATAAAGTTGATATTAAAAATGAAAATAGTTCACATTGTAATGATTTAGGAAGTACAGATGAAGGTGATAAAACA

TTATGTAAGAAAATAGTTCAAAATTTAAATCAATTAAGTGCATTAAAAGATGATGAAAATTTAGATAATTCATGT

TATTATTTTCAACATTGGTTTTTCGATAATATAGCTAAAAAATATTATGATGGAGATGAAAGAGGTAATAATTAT

CCAGTTGCAGAAAAATTGTATAATATTGTTAGTGAAATTACACCAGTAAGTTCAAAAATGGAACCATGTAAATGT

TATGAATCAGGATATCCAGATGTATGGAAAGAAGAAAAACATTTACATGATTATTTTGAAAATCATAAAGATATA

AAATGTAATGATAGTGATAAATCAAAATGTGAAAAATATATACAATATGTTACATATATTAATACATTATTTCAA

GAAAAAGTAGATAAATGTTGTGATGGAGAAGATTTAGATGAATATGGTTTTTGTGAACCATATTTTAAATGTGAA

AATAAATATTCACCACAAGATTTATTAGCACAATTAAAGAAAGAATTACAAGAATTAGGAAAGAAAGCAGAAGCT

CCAAGAGATGGAGGTACAGGAGCAGTTGAAGCTGGTAAAGCACCAGGAAGTGGTGCTGAAGAAAGAGAAAAGAAA

ACAAAAGAAGAAGTAACAGCAGAAGCTACAGGAAAAGAATCAAAAGAAAAATTGGTTGCAGGTGAAGTACCAAAA

GAAAAAGCAGCTGATAGTTTAGCTCAATCAGCATTAGAAAAAACAATACCAACACAACCAGCTGTTCAAGGAAGT

GTAGGTTTAAAACCAGGAGAAAGTGAATCAAGAGAAGAACCAGCATCAGCTAAATTGGTTGGTAAAAAACCACCA

GAAGAAAATCCAATTGCACCAGAAAGTGGAGGTGCTAAACCAGCACCATCAGAAGGAGCACCAGCTAAACCAGTT

GCTACAAAACCACCAGCAGCTGGTCCAGTTGCAGTAGAAAGTGAAACAGTAACACCAGCAATGGCTGAATCAGGA

AAAGCTAAATTGAGAGAAACAAAACCTGCTCCAGCTAAACCAGTTGCAGCTAAACCAGTAGCTAGTAAACCAGAA

ACAGAAAAAGCAAAAGAAATTGTTGCTGAAAAAGAAGCAACAAAAGAAATTGTAGCTGAAGAAGAAGCAACTAAA

GAAACTTTAGTAGAAGAAGAAGCACCAGAAGAAGTTGTAGATGAAACAACAAATACATTACAACCAGAAGAACCA

TTAGGACCACCACCAATAGAAAGTGCTGAACAAGATGTTCATGTAATTAGTTCACATAATACAGAACCAGCTTCA

AATGCATTACCATTAACAATTACAGATACACCAAATACATTAGGAACAGCACATGAAGGTTTAGATAGTAATTTC

TTTAGAAATATAATAATGGCAGTTGCTGTATTAGGTAAAATATTTTTCTTATTTTATTATAATAGAAGTTCAAGA

TTAGAAAGTTCATTACGTAAAAAGAAAAGAAAGAAAGGAAAAATATTCGAACATAATTATTATGAAGAATATGAA

AAAGAATTAGAAATGTATGGTTCAGAAGAAACATTTTTAGATTCAGAAACAGATAGATTATATTTAAATTATCAT

CCAGATCAAGATAGTTATTAT

CCTAGG

PVX_108770

```
            M  F  D  L  E  G  G  G  T  Q  V  E  A  D  D  E  T  I  S  E  S
1.    GGTACCATGTTTGATTTAGAAGGTGGTGGAACACAAGTAGAAGCAGATGATGAAACAATATCAGAAAGT
      T  I  Q  G  Q  R  I  I  F  E  H  L  P  A  Y  I  F  E  Q  K  L  K  E
70.   ACAATTCAAGGACAAAGAATTATATTTGAACATTTACCAGCTTATATTTTTGAACAAAAATTAAAAGAA
      D  A  T  D  N  N  F  S  G  Y  Y  N  V  V  K  H  I  S  E  R  Y  G  W
139.  GATGCAACAGATAATAATTTTTCAGGATATTATAATGTAGTAAAACATATATCTGAAAGATATGGATGG
      G  N  D  L  F  K  K  L  S  R  N  I  S  L  V  H  D  S  Y  I  E  G  D
208.  GGAAATGATTTATTTAAAAAATTATCAAGAAATATTAGTTTAGTACATGATAGTTATATTGAAGGGGAT
      E  F  N  R  K  R  C  Y  D  L  N  Y  W  L  Y  D  N  V  Y  K  N  L  E
277.  GAATTTAATAGAAAAAGATGTTATGATTTAAATTATTGGTTATATGATAATGTTTATAAAAATTTAGAA
      S  S  N  T  N  D  T  D  Y  F  K  D  I  S  T  K  L  Q  G  V  W  K  N
346.  AGTAGTAATACAAATGATACAGATTATTTTAAAGATATATCAACAAAATTACAAGGAGTATGGAAAAAT
      I  V  D  N  E  F  K  D  R  P  Y  Q  C  Y  P  D  K  E  L  L  L  N  M
415.  ATAGTAGATAATGAATTTAAAGATAGACCATATCAATGTTATCCAGATAAAGAATTATTATTAAATATG
      G  Y  L  Q  E  I  K  D  L  F  D  F  Y  E  D  Y  N  E  M  K  K  E  I
484.  GGATATTTACAAGAAATAAAAGATTTATTTGATTTTTATGAAGATTATAATGAAATGAAAAAAGAAATT
      I  A  D  T  S  G  S  C  R  R  Y  V  E  Y  L  K  Q  R  I  P  V  Y  Y
553.  ATAGCAGATACAAGTGGAAGTTGTAGAAGATATGTAGAATATTTAAAACAAAGAATACCAGTATATTAT
      T  W  R  D  S  C  K  V  P  E  Y  T  C  K  R  Y  I  D  D  Y  M  K  Y
622.  ACTTGGAGAGATAGTTGTAAAGTACCAGAATATACATGTAAAAGATATATTGATGATTATATGAAATAT
      R  P  A  S  I  V  P  D  L  S  P  W  V  V  L  T  Y  P  G  N  K  C  Y
691.  AGACCAGCAAGTATAGTACCAGATTTAAGTCCATGGGTAGTATTAACATATCCTGGAAATAAATGTTAT
      A  T  V  Y  D  I  F  V  K  A  K  E  Q  P  K  R  N  D  G  I  Y  K  K
760.  GCAACAGTATATGATATTTTTGTAAAAGCAAAAGAACAACCTAAAAGAAATGATGGAATTTATAAAAAA
      K  M  E  K  L  E  K  L  N  P  G  K  S  L  L  N  I  S  M  G  E  G  L
829.  AAAATGGAAAAATTAGAAAAATTAAATCCTGGAAAAAGTTTATTAAATATTAGTATGGGAGAAGGATTA
      R  G  S  E  F  F  I  P  G  D  H  D  D  Y  W  L  R  L  K  W  D  I  L
898.  AGAGGAAGTGAATTTTTTATTCCTGGTGATCATGATGATTATTGGTTAAGATTAAAATGGGATATATTA
      L  Y  I  T  D  D  I  T  P  P  V  L  G  I  V  G  T  L  L  I  F  W  A
967.  TTATATATTACAGATGATATTACACCACCAGTATTAGGAATAGTAGGAACATTATTAATATTTTGGGCA
      L  Y  K  V  R  K  R  I  R  P  N  I  N  Y  D  D  I  K  L  L  Y  G  S
1036. TTATATAAAGTTAGAAAAAGAATTAGACCAAATATTAATTATGATGATATTAAATTATTATATGGAAGT
      E  E  S  L  N  S  S  T  D  S  Y  D  Y  N  N  D  Y  D  N  D  Y  D  N
1105. GAAGAAAGTTTAAATAGTTCAACAGATAGTTATGATTATAATAATGATTATGACAATGATTATGATAAT
      D  D  Y  S  D  S  N  S  S  Y  N  L  S  Y  A  S  T  L  D  Y
1174. GATGATTATAGTGATAGTAATAGTAGTTATAATTTAAGTTATGCAAGTACATTAGATTATCCTAGG
```
