# Supplementary figures and images for: Ectopic Expression of Plasmodium vivax vir Genes in P. falciparum Affects Cytoadhesion via Increased Expression of Specific var Genes
Source: Microorganisms. 2022 Jun 9;10(6):1183. doi: 10.3390/microorganisms10061183 (PMC9230084; doi:10.3390/microorganisms10061183)

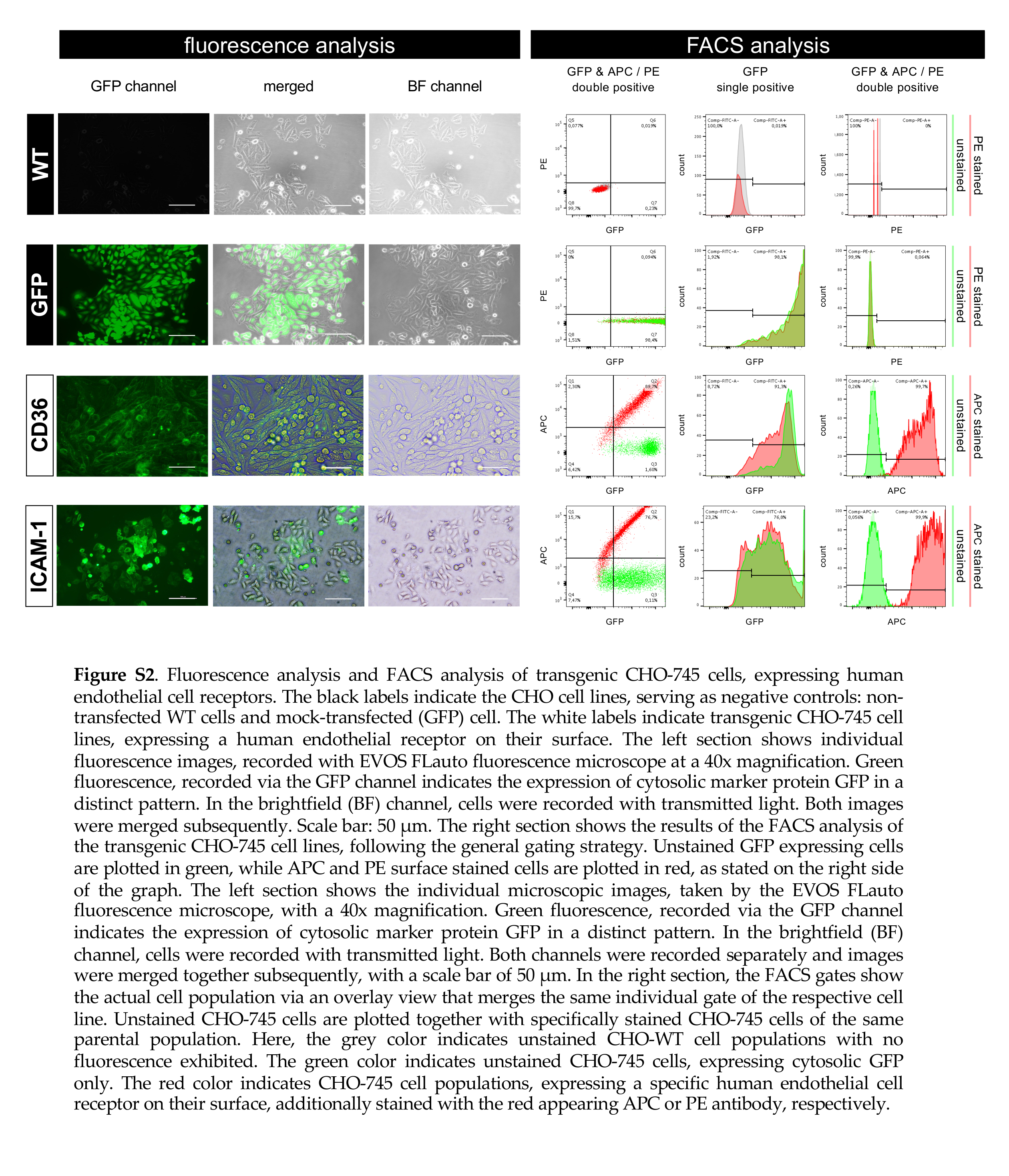

Supplement: Supplementary file 1 [file microorganisms-10-01183-s001.zip › Figure S2.tif]

# PVX\_050690

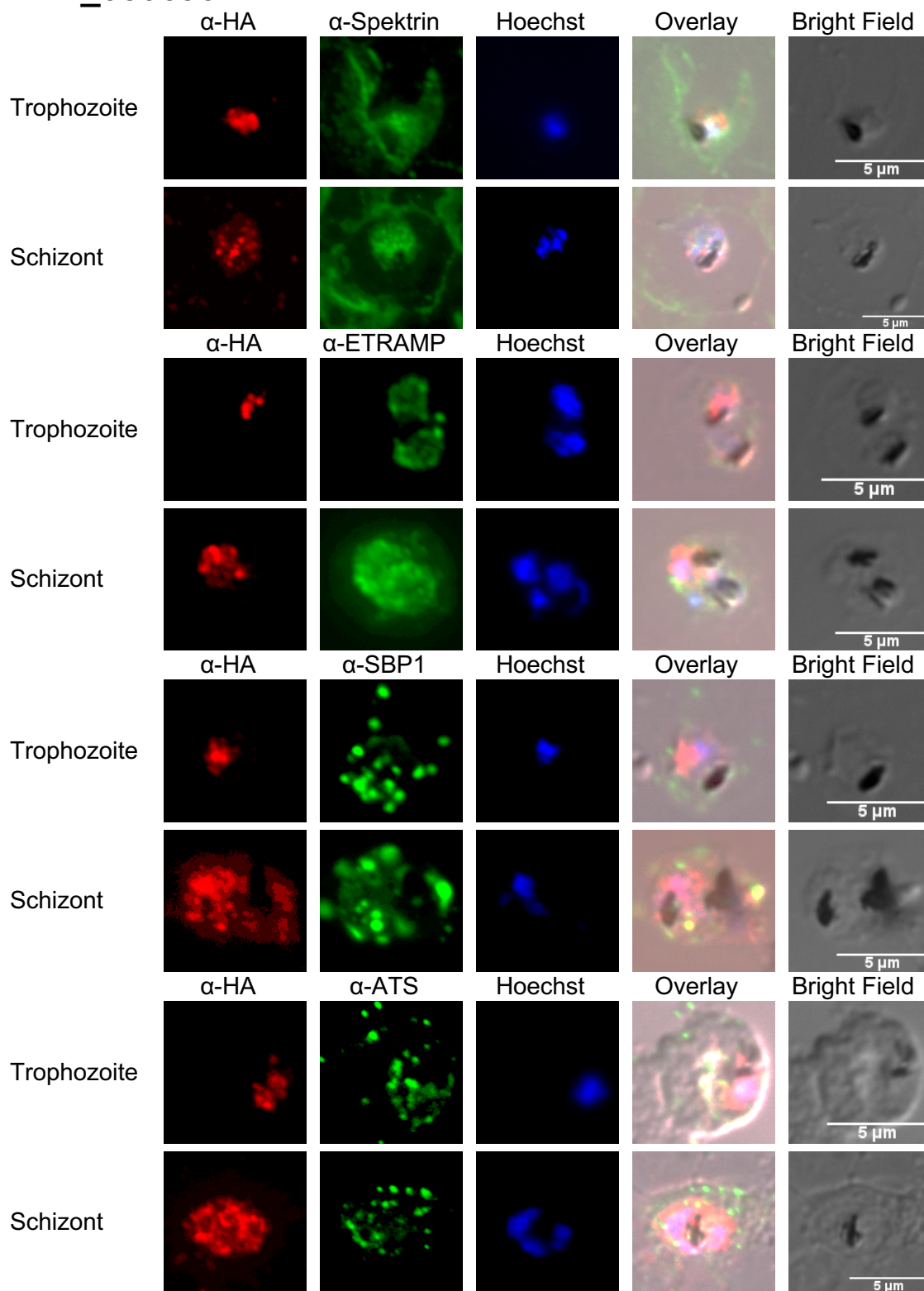

PVX\_060690

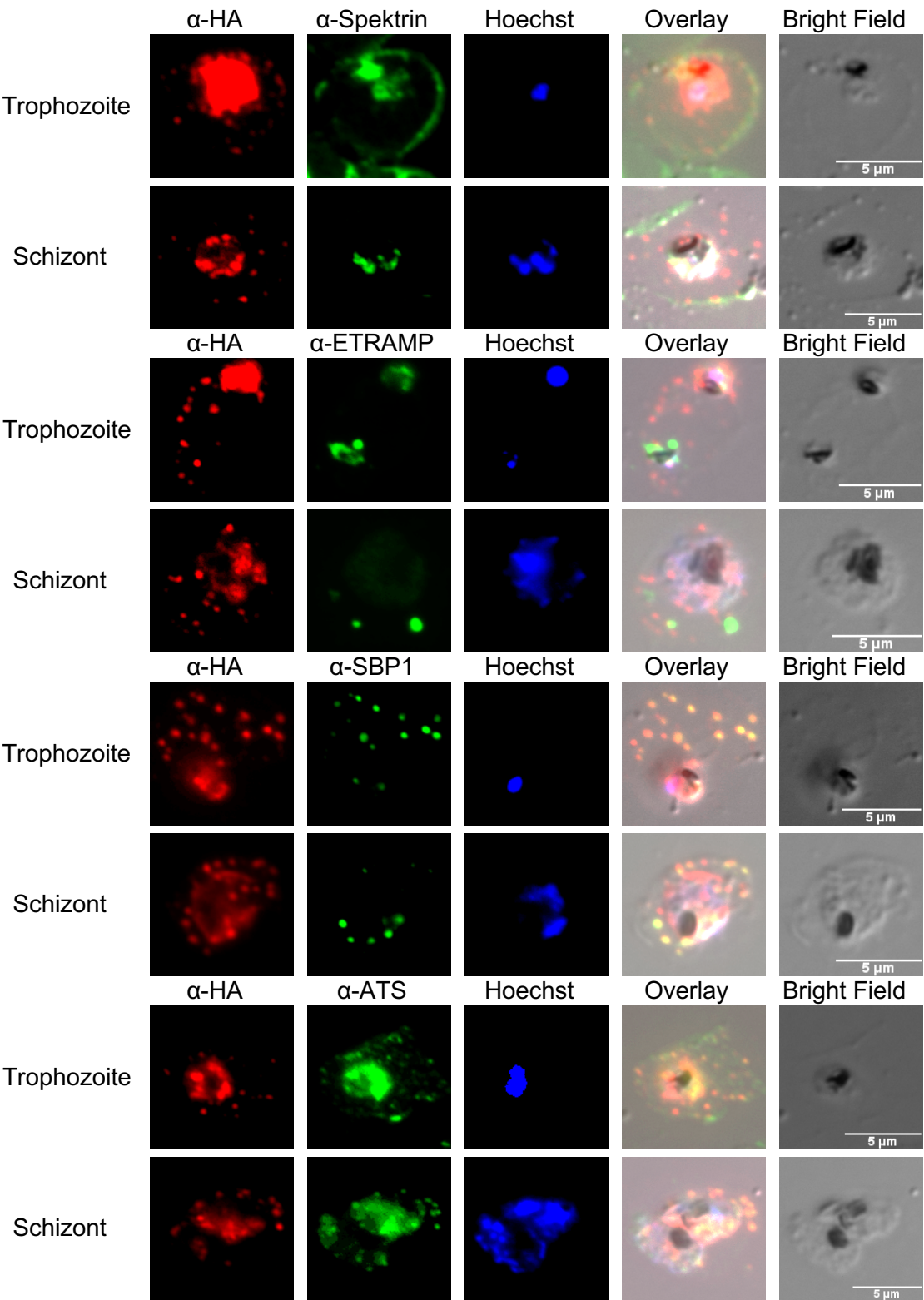

**PVX\_068690**

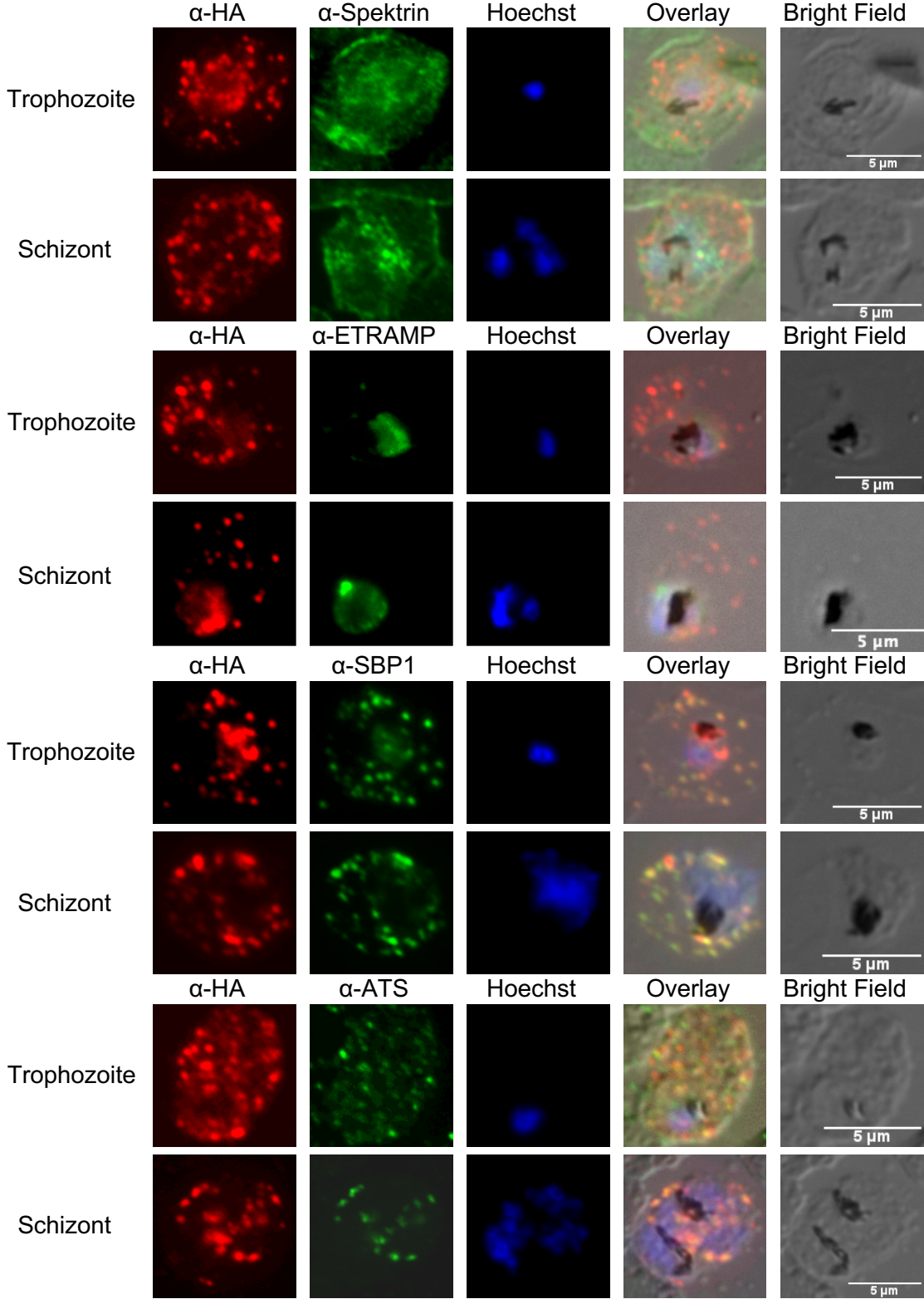

**PVX\_093715**

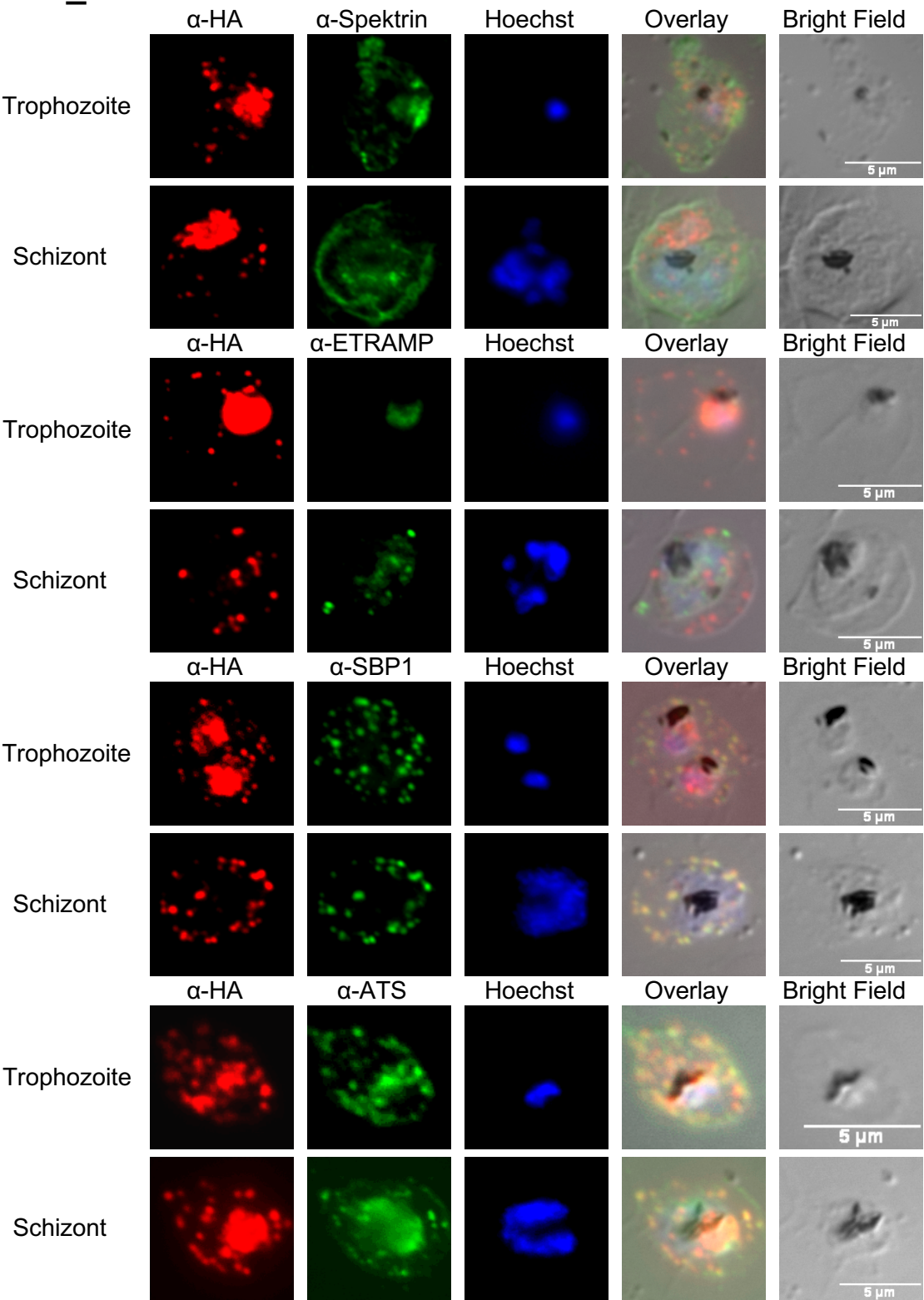

PVX\_096925

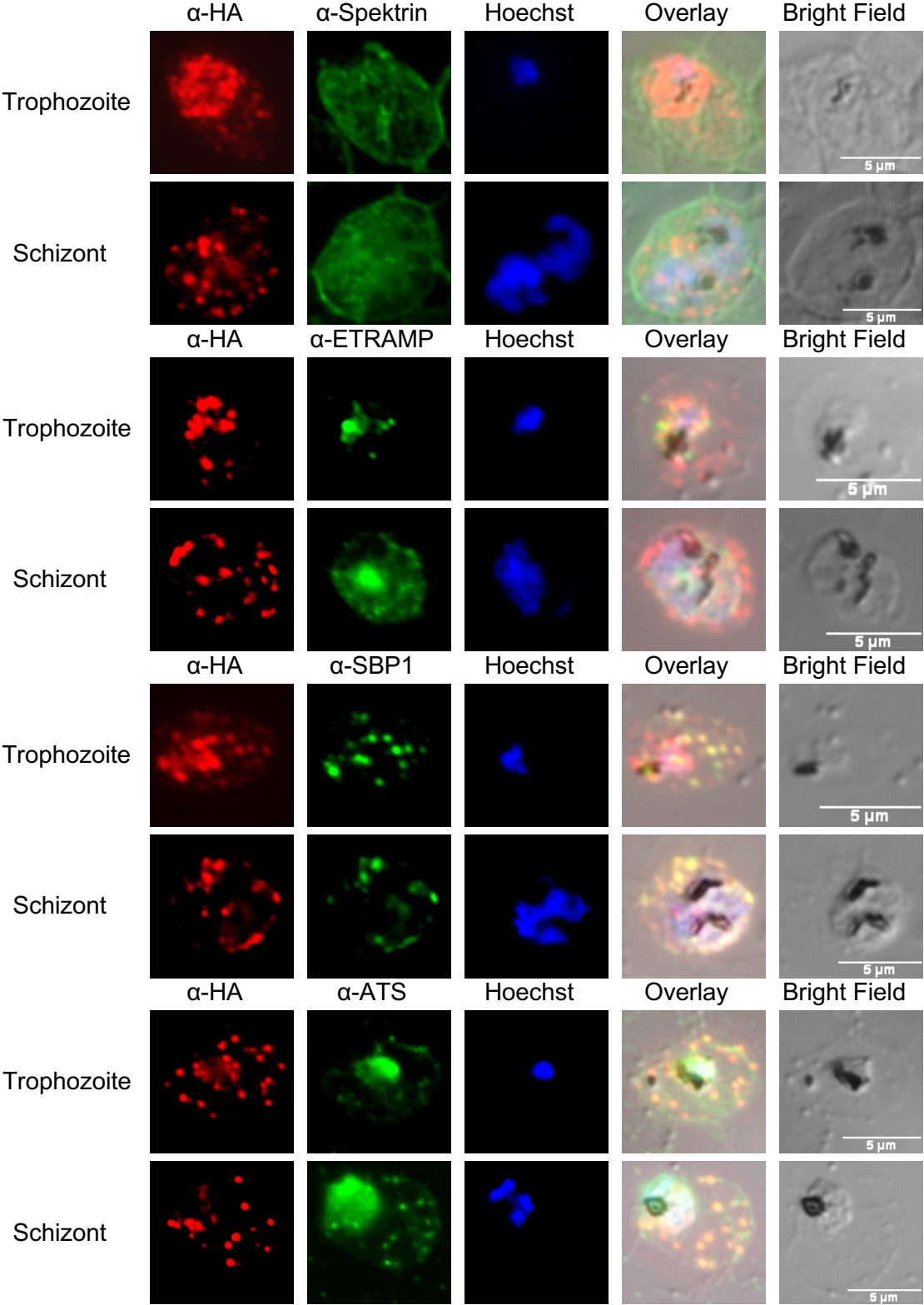

PVX\_077695

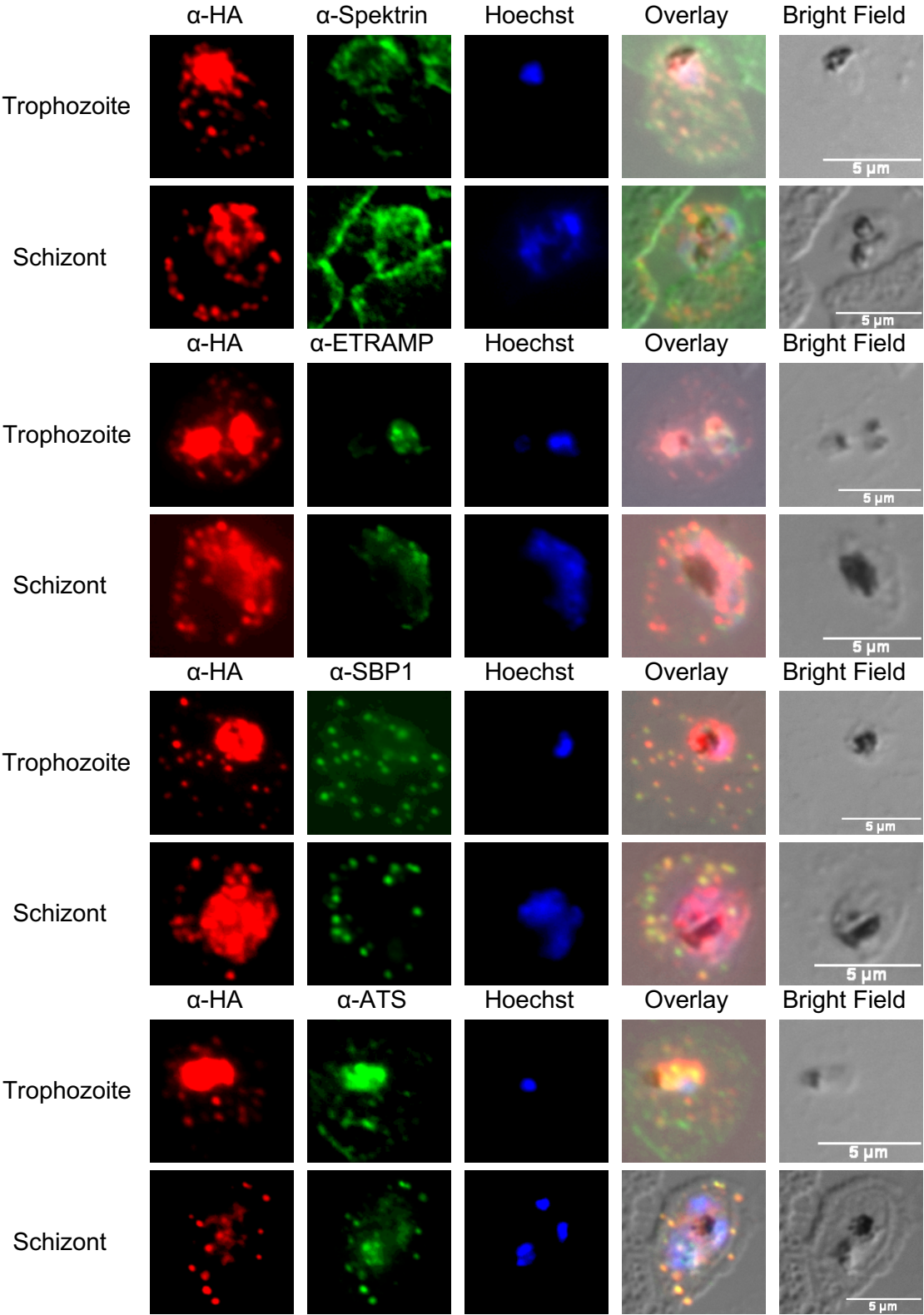

**PVX\_081850**

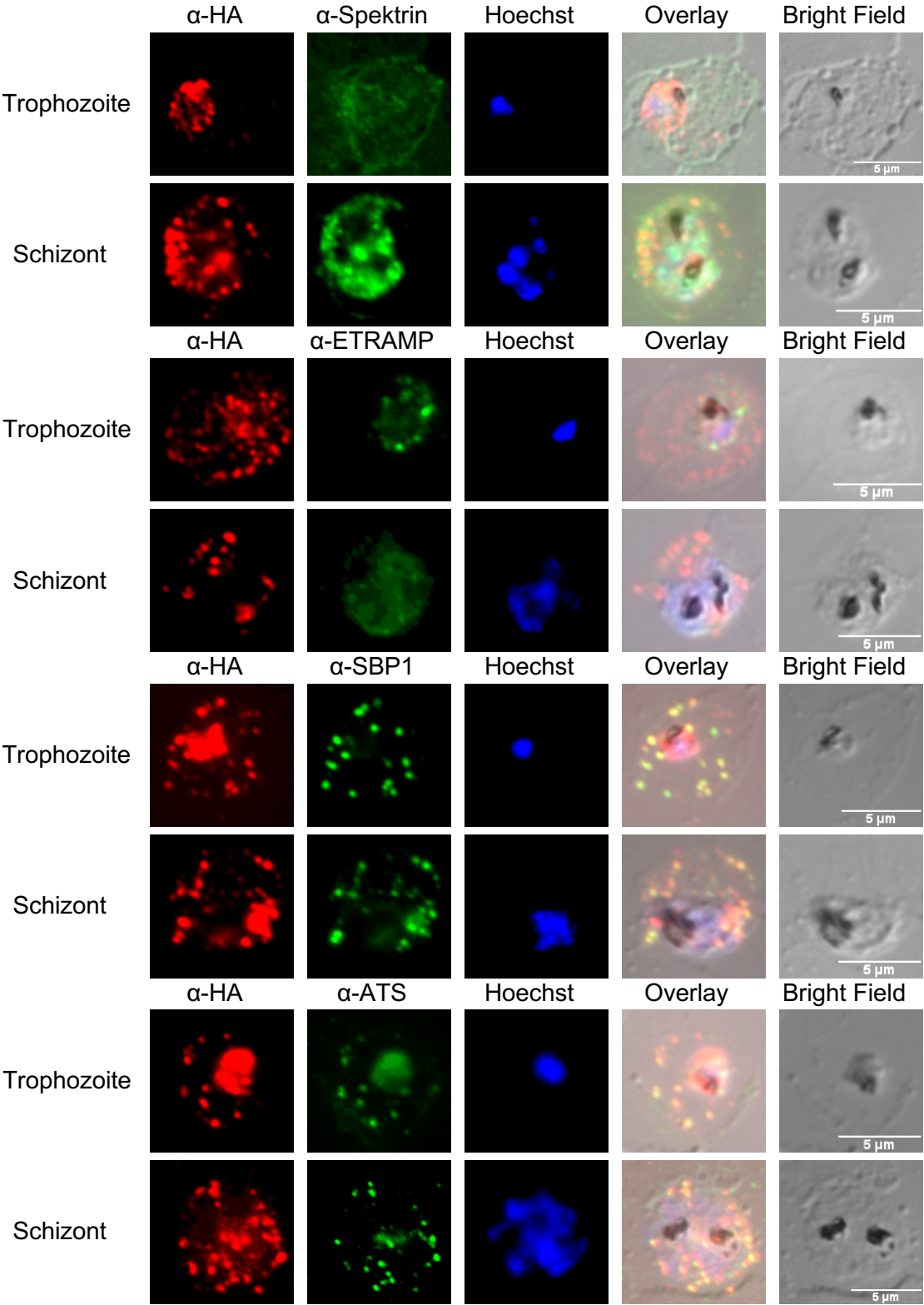

PVX\_097525

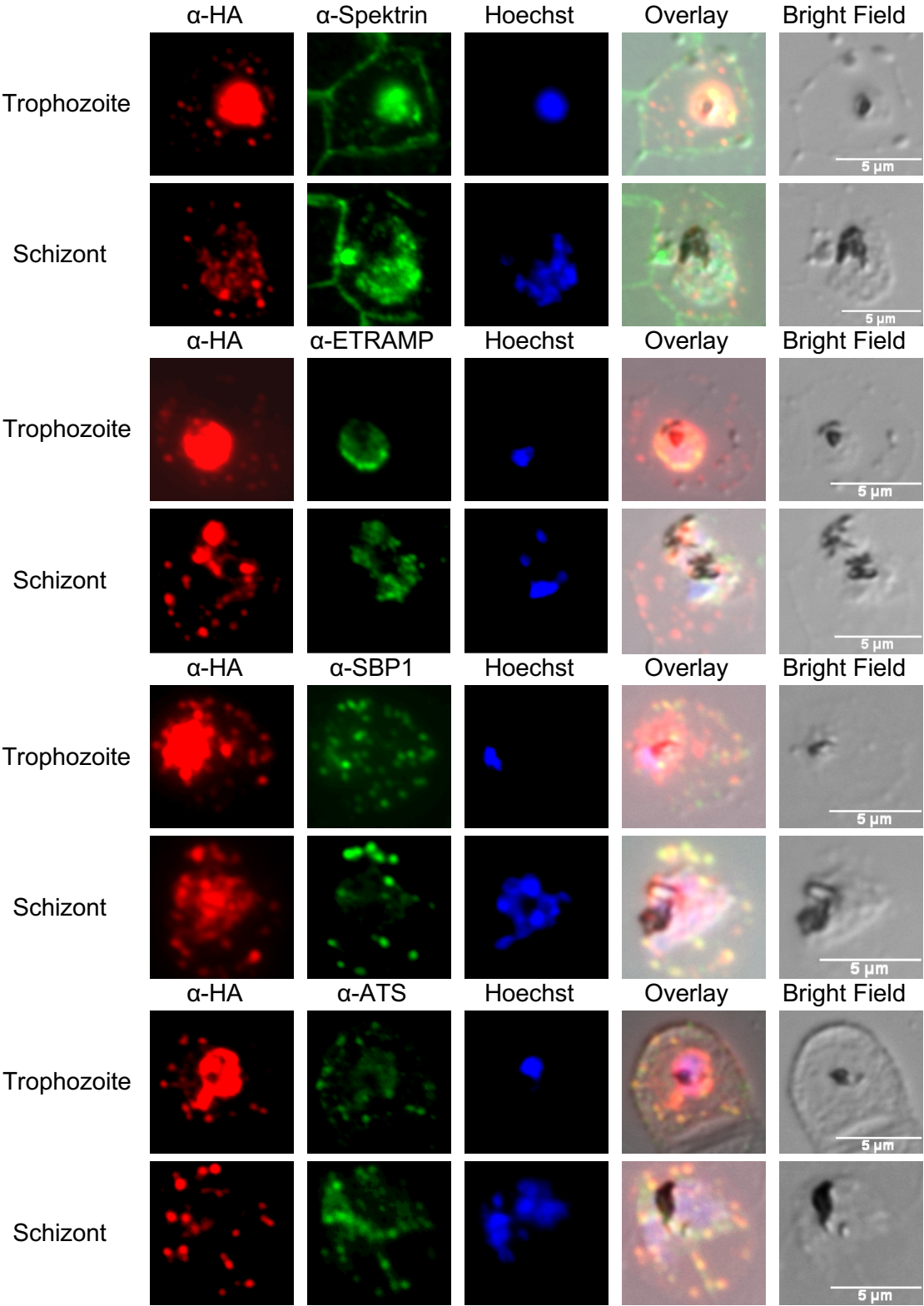

**PVX\_101560**

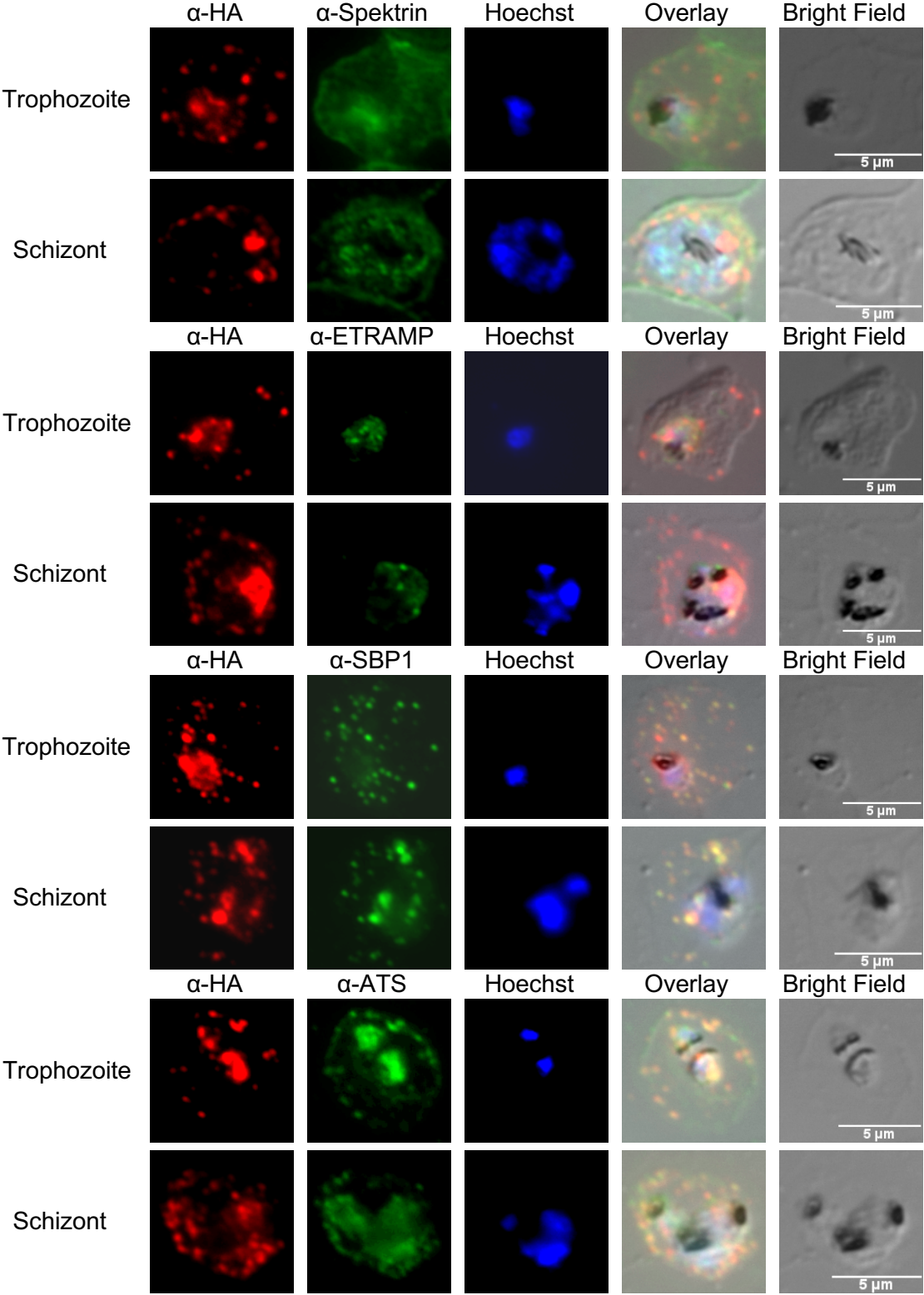

**PVX\_107235**

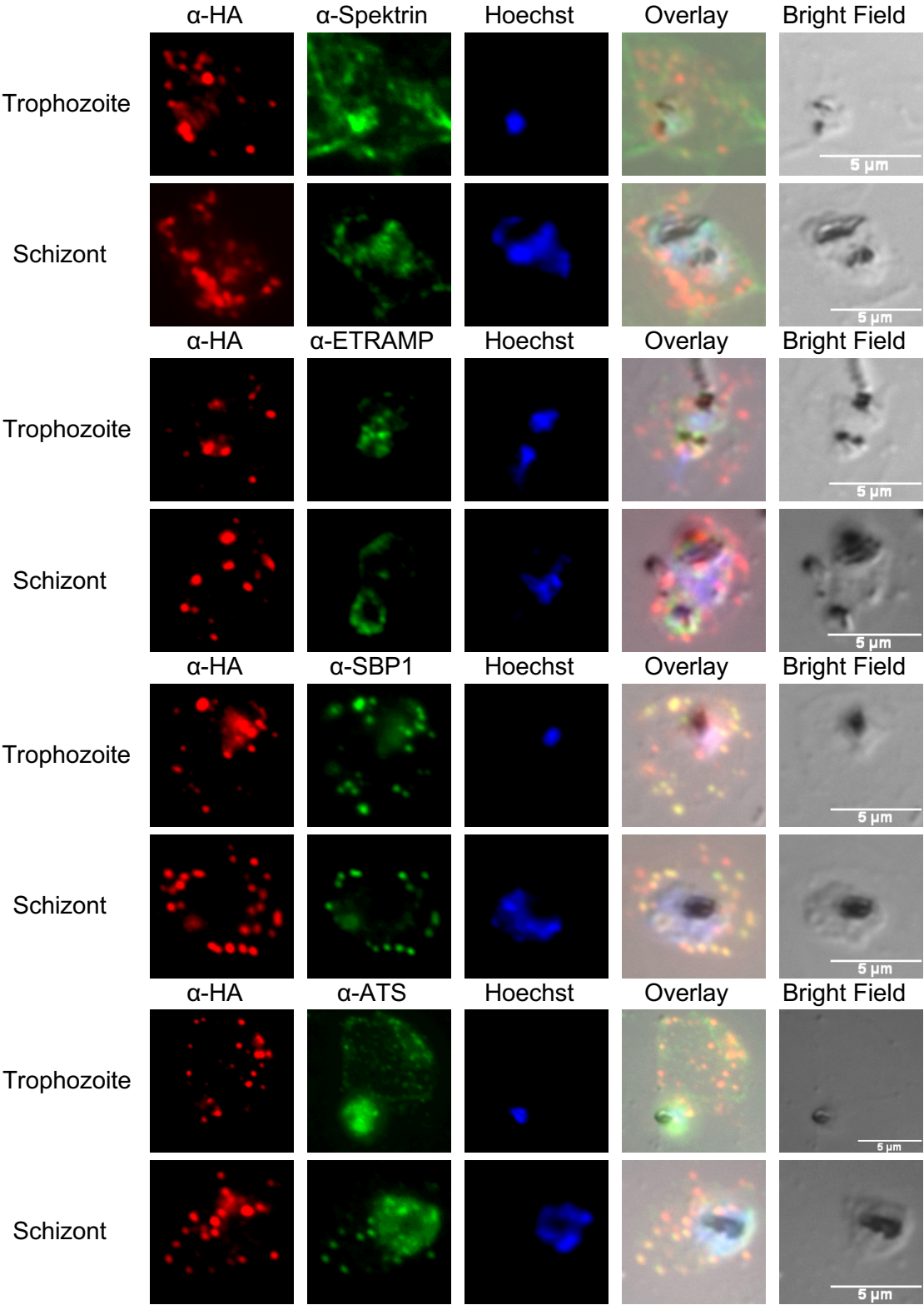

**PVX\_113230**

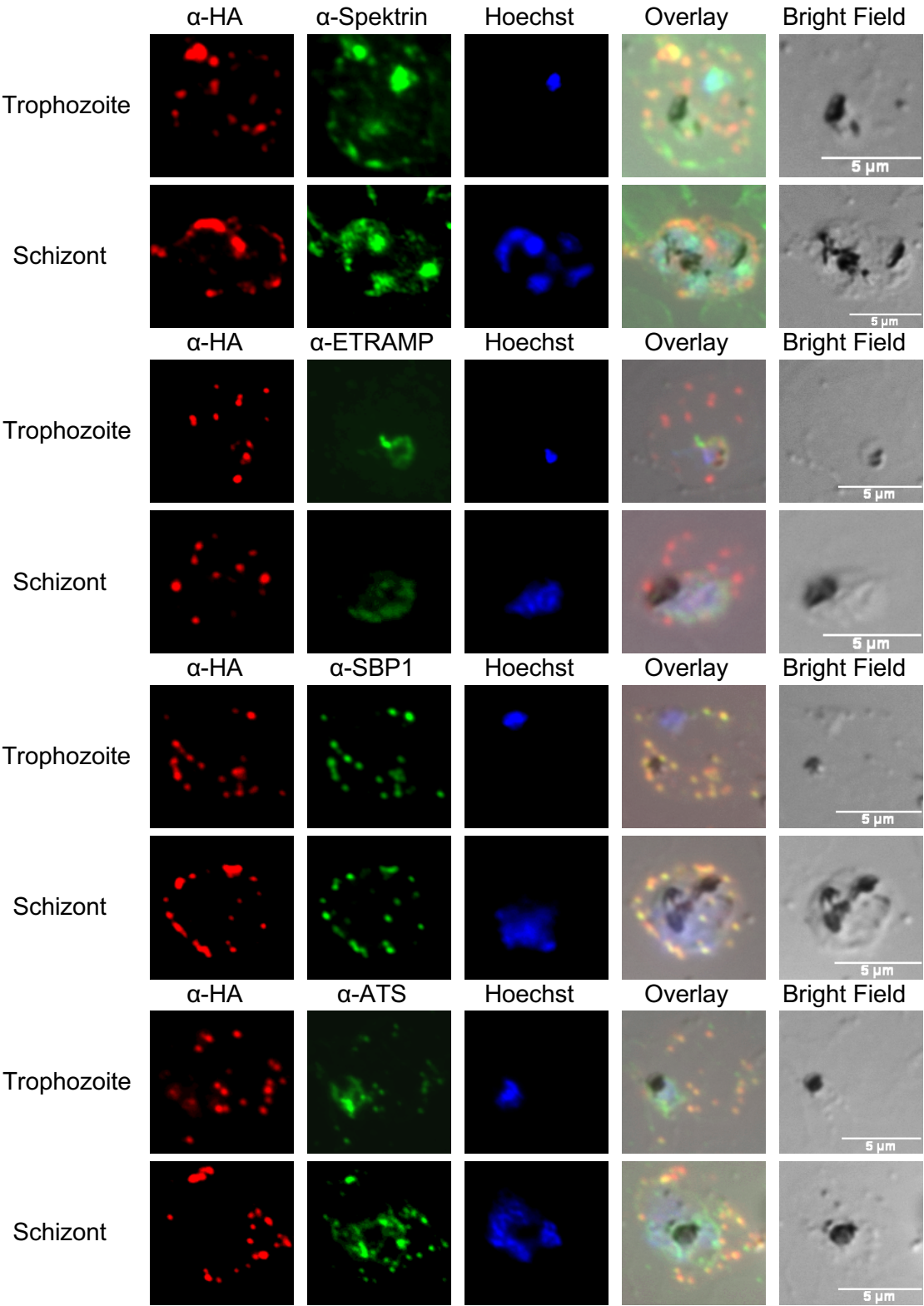

**PVX\_115475**

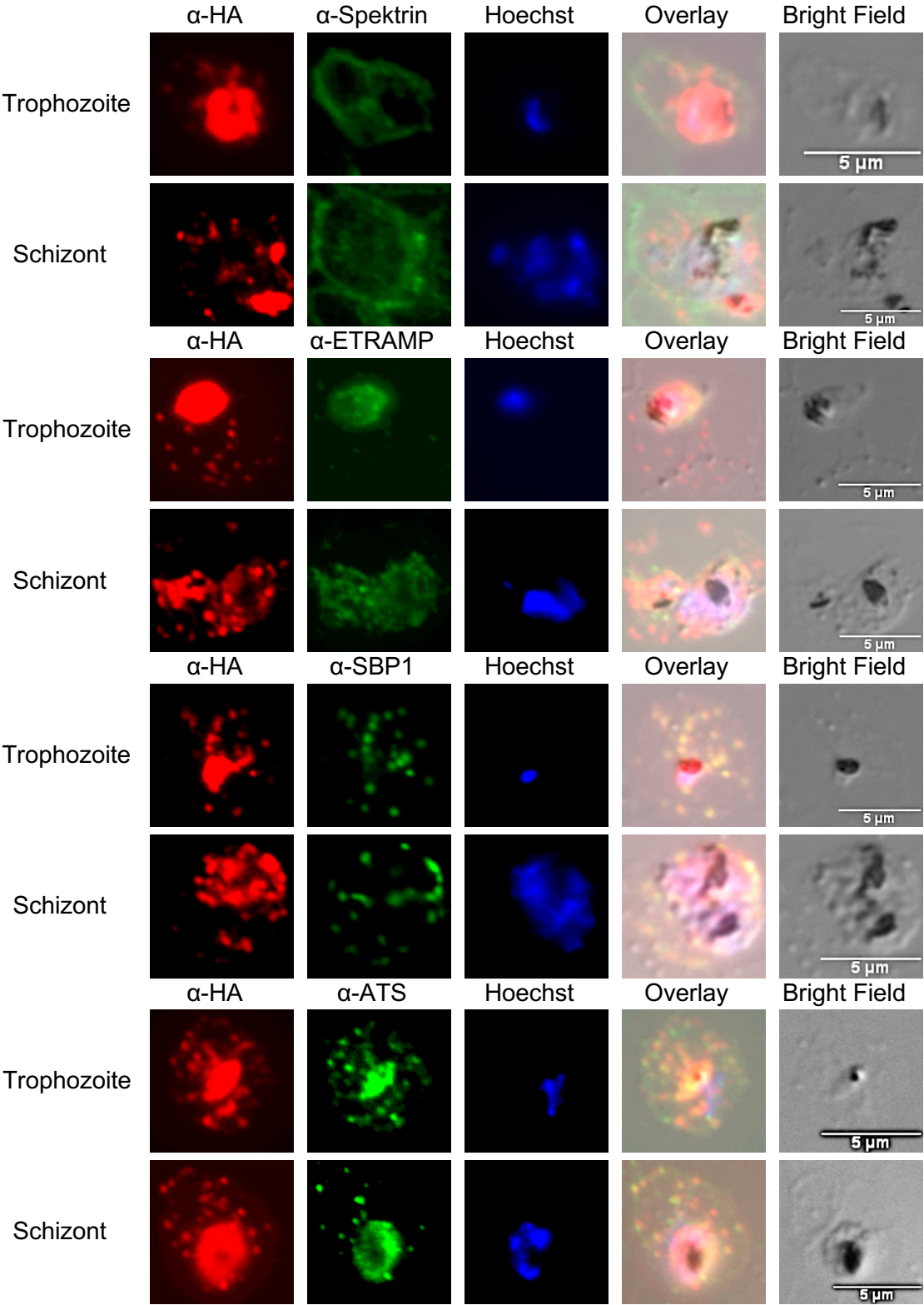

Supplement: Supplementary file 1 [file microorganisms-10-01183-s001.zip › Figure S3.pdf]
